# Antimicrobial and Seasonal Evaluation of the Carvacrol-Chemotype Oil from *Lippia origanoides* Kunth.

**DOI:** 10.3390/molecules20021860

**Published:** 2015-01-23

**Authors:** Sandra Layse F. Sarrazin, Leomara Andrade da Silva, Ana Paula F. de Assunção, Ricardo B. Oliveira, Victor Y. P. Calao, Rodrigo da Silva, Elena E. Stashenko, José Guilherme S. Maia, Rosa Helena V. Mourão

**Affiliations:** 1 Programa de Pós-Graduação em Biodiversidade e Biotecnologia da Amazônia Legal, Universidade Federal do Amazonas, 60077-000 Manaus, Brazil; E-Mail: sarrazin@ufpa.br; 2 Programa de Pós-Graduação em Recursos Naturais da Amazônia, Universidade Federal do Oeste do Pará, 68135-110 Santarém, Brazil; E-Mails: Andrade.biologia@hotmail.com (L.A.S.); anapaula_itb@hotmail.com (A.P.F.A.); rbo@ufpa.br (R.B.O.); vyperez@gmail.com (V.Y.P.C.); rsilvf@yahoo.com.br (R.S.); 3 Chromatography Laboratory, Research Center for Biomolecules, Industrial University of Santander, Bucaramanga 57, Colombia; E-Mail: elena@tucan.uis.edu.co; 4 Programa de Pós-Graduação em Química, Instituto de Ciências Exatas e Naturais, Universidade Federal do Pará, 66075-110 Belém, Brazil

**Keywords:** *Lippia origanoides*, Verbenaceae, salva-do-marajó, essential oil composition, seasonal variation, antimicrobial evaluation

## Abstract

This study evaluated the influence of seasonal variation on the yield and composition of essential oil of *Lippia origanoides* occurring in the Middle Rio Amazonas, Brazil, and the impact on its antimicrobial potential. The average oil yield was 1.7% ± 0.2% in the rainy season and 1.6% ± 0.3% in the dry season. Some correlations with climatic parameters were observed. The major components were carvacrol (rainy, 43.5% ± 1.9%; dry, 41.4% ± 2.04%), thymol (rainy, 10.7% ± 1.1%; dry, 10.6% ± 0.9%), *p*-cymene (rainy, 9.8% ± 0.7%; dry, 10.0% ± 1.4%) and *p*-methoxythymol (rainy, 9.6% ± 0.8%; dry, 10.4% ± 1.4%). It was found that the antibacterial activity of *L. origanoides* against *Staphylococcus aureus* and *Escherichia coli* was little influenced by the changes in oil composition due to seasonal variation. Against *S. aureus*, the oil Minimum Inhibitory Concentration (MIC) value was 1.25 μL/mL over ten months. Against *E. coli*, the oil MIC values ranged from 0.15 μL/mL to 0.31 μL/mL in different months of the year. The Minimum Bactericidal Concentration (MBC) value was 2.5 μL/mL against *S. aureus* and 1.25 μL/mL against *E. coli*. The results suggest that the antimicrobial activity identified in the oil remain unchanged for the full year, allowing its medicinal use without any risk of loss or absence of the active principles of the plant.

## 1. Introduction

*Lippia origanoides* Kunth, known in northern Brazil as “salva-do-marajó” and “alecrim-d’angola”, is a shrubby species with a perennial life cycle, whose aerial parts are used in cooking as a flavoring of regional dishes, in the treatment of gastrointestinal disorders, respiratory diseases, and as an antiseptic for mouth and throat irritation. This species is also called oregano in Mexico and used as a substitute for *L. graveolens* Kunth and *Origanum vulgare* L., the Mexican and Greek oreganos, respectively [1,2,3].

The production of chemical components in plant species is influenced by external factors such as soil quality and climatic conditions. The yield of an essential oil and its composition are susceptible to quantitative and qualitative variations and consequently the biological activity of this oil is dependent on its composition and similarly will be subject to variations in its potency [4,5]. Thus, plants sampled in different seasons may have different compositions and consequently contain or not specific bioactive constituents [6].

The oil composition of *L. origanoides* occurring in the Brazilian and Colombian Amazon has shown significant variation based on its main constituents. It is represented by different chemical types as already described: *p*-cymene, α- and β-phellandrene and limonene (chemotype A) [7], carvacrol (chemotype B) [2,3], thymol (chemotype C) [7,8], 1,8-cineole (chemotype D) [9], and lately (*E*)-methyl cinnamate and (*E*)-nerolidol (chemotype E) [10].

The bioactivity of the essential oils produced from *L. origanoides* has been evaluated. They showed antimicrobial activity against some pathogenic microorganisms [2,3,11,12,13,14], repellent activity against pests living in stored grains such as *Sitophilus zeamais* and *Tribolium castaneum* [15,16], an inhibitory effect on yellow fever virus replication *in vitro* [17], antiprotozoal activity against *Leishmania chagasi* and *Trypanosoma cruzi* [18,19], and DNA protective effects against bleomycin-induced genotoxicity [20]. The composition of the oil of *L. origanoides* and its antioxidant activity were analyzed after cultivation and mineral fertilization [21]. These qualities give to the plant a biological potential for its use as medicine in the control of some infectious diseases, in association to its essential oil that also possesses important properties of significant interest in the cosmetics and food markets.

The present study investigated for one year the influence of seasonal variation on the yield and composition of essential oil from fresh leaves and thin stems of *L. origanoides* collected in the municipality of Santarém, Pará State, as well as the variation in the antimicrobial potential of these oils, in order to obtain biological data that would allow a better utilization of the plant for therapeutic and medicinal purposes.

## 2. Results and Discussion

### 2.1. Effect of Seasonal Variation in Oil Yield

**Figure 1:**
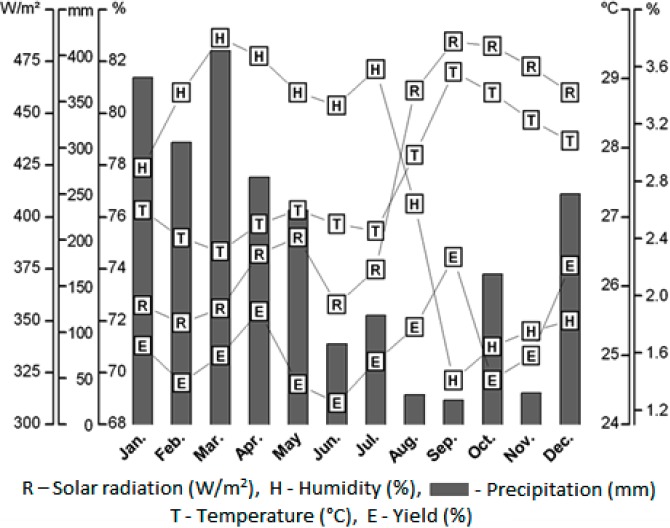
Yields (%) of the essential oil of *L. origanoides* and climatic variables measured at the time of collection: precipitation (mm); solar radiation (W/m^2^); relative humidity (%) and temperature (°C).

Parameters such as temperature, solar radiation, precipitation and air relative humidity were measured during the sampling period of *L. origanoides* to verify the possible influence of seasonal variation in the oil yield. The results are shown in Figure 1.

The Brazil Northern region is characterized by two distinct seasons: the rainy season, between December and May, and the dry season, between June and November. In 2012, the average values of temperature and solar radiation, between January and December, ranged from 26.44 °C to 29.06 °C and 357.86 W/m² to 484.59 W/m², respectively. The average relative humidity and the precipitation ranged from 69.69% to 82.88% and from 26.2 mm to 405.3 mm, respectively. The observed precipitation in January, February and March, was higher when compared to the other months of the year. On the other hand, the temperature remained almost constant, with a minimum in March (26.44 °C) and maximum in September (29.06 °C).

The oil yield ranged from 1.3% to 2.3% over twelve months, with a mean of 1.7% ± 0.2% in the rainy season and 1.6% ± 0.3% in the dry season. The month of sampling did not affect the average oil yield (*p* > 0.05). These results are consistent with studies published by Rojas and colleagues [8], who also observed an average yield of 1.1% for the oil of *L. origanoides* collected in Merida, Venezuela, in both seasons. In this study, correlations were observed when comparing the oil yields and climatic parameters measured during the collection of the plant. The oil yield was directly proportional to increased solar radiation and temperature and inversely proportional to the air relative humidity rate (see Table 1). According to Castelo and colleagues [22], essential oils play an important role in plant defense against harsh environmental conditions. For example, high temperatures can promote change in the activity of the stomata of leaves, thus reducing volatilization of these oils.

**Table 1:** Correlation between the oil yield of *L. origanoides* with the solar radiation, temperature and relative humidity, in the collection area.

Environmental Factors	Correlation Coefficient (r^2^)
Temperature (°C)	0.49 *
Air relative humidity (%)	−0.49 *
Radiation (W/m²)	0.51 **

* Significant at *p* ≤ 0.1; ** Significant at *p* ≤ 0.05.

### 2.2. Effect of Seasonal Variation in Oil Composition

The oils extracted from the plants collected in the period from January to December 2012 were analyzed by GC and GC-MS. Individual components were identified by comparison of both mass spectrum and GC retention data with authentic compounds, which were previously analyzed and stored in the data system, as well as with the aid of commercial libraries containing retention indices and mass spectra of volatile compounds, commonly found in essential oils [23,24]. The identified constituents are listed in Table 2.

Thirty-seven constituents were identified in the twelve analyzed oils, accounting for an average of 92% to 99% of the total. According to seasonal period, the main constituents were carvacrol (rainy season, 43.5% ± 1.9%, dry season, 41.4% ± 2.04%, *p* < 0.05), thymol (rainy season, 10.7% ± 1.1%, dry season, 10.6% ± 0.9%, *p* > 0.05), *p*-cymene (rainy season, 9.8% ± 0.7%, dry season, 10.0% ± 1.4%, *p* > 0.05) and *p*-methoxythymol (rainy season, 9.6% ± 0.8%, dry season, 10.4% ± 1.4% , *p* < 0.05). The amount of carvacrol, the major component, in the oils of the two seasons showed a small difference, about 2%, with no statistical significance. The four major components (carvacrol, thymol, *p*-cymene and *p*-methoxythymol) showed a higher percentage in April, with a total of 78.6%. Some constituents with low-percentage content were shown to be present or absent according to the month of collection. In addition, a greater number of components was identified in the oil of May 2012.

Differences in essential oil composition under the influence of phenological stage or environmental factors have been reported. For example, in *Satureja* species from the Mediterranean region, carvacrol was the main component during the flowering period, and thymol for most of the year except winter, where γ-terpinene showed predominance [25]. Thus, the phenological stage and environmental factors can change in a significant manner the biochemical pathways and physiological processes that alter the plant metabolism and, therefore, the essential oil biosynthesis [26,27].

The genus *Lippia*, with more than fifty reported essential oils, is well known for its aromatic character [1,28]. Furthermore, the *Lippia* species that occur in Brazil have shown a wide variation in the oil composition with the description of several chemotypes, such as *Lippia alba* (Mill.) N.E. Br collected in Pará and Ceará with citral, carvone and 1,8-cineole types [29,30]; *Lippia lupulina* Cham. existing in Mato Grosso state with terpinen-4-ol, 1,8-cineole + β-caryophyllene, and germacrene D + β-caryophyllene + bicyclogermacrene types [31]; *Lippia grandis* Schauer with occurrence in all Amazon Region with thymol, 1,8-cineole and carvacrol types [32,33]; and *Lippia glandulosa* Schauer that grows in Roraima with thymol and β-caryophyllene types [34].

**Table 2:** Yield and essential oil composition of *L. origanoides* collected from January to December 2012.

Month/Oil Yield			Jan	Feb	Mar	Apr	May	Jun	Jul	Aug	Sep	Oct	Nov	Dec
			1, 7 *	1, 4 *	1, 6 *	1, 9 *	1, 4 *	1, 3 *	1, 5	1, 8	2, 3	1, 4	1, 6 *	2, 2 *
Constituents	RI_Calc._	RI_Lit._	Oil %											
(*Z)*-Hexen-3-ol	854	859	-	0.2	0.4	-	0.2	0.2	0.2	0.3	0.3	-	-	0.3
α-Thujene	926	924	0.7	0.6	0.7	0.7	0.5	0.6	0.8	0.7	0.7	0.8	0.9	0.6
α-Pinene	934	932	0.6	0.4	0.4	0.4	0.3	0.4	0.4	0.5	0.5	0.6	0.7	0.5
1-Octen-3-ol	976	974	0.1	0.2	0.3	0.1	0.3	0.1	0.2	0.2	-	-	-	-
β-Pinene	978	976	0.2	0.2	0.1	-	0.1	-	-	0.2	0.2	0.2	0.3	0.2
Myrcene	990	988	1.3	1.2	1.3	1.3	1.0	1.1	1.2	1.1	1.2	1.5	1.5	1.1
α-Terpinene	1016	1014	0.7	0.8	0.9	0.9	0.7	0.5	0.7	0.7	0.5	0.7	0.8	0.7
*p*-Cymene	1025	1020	11.2	9.6	10.5	10.2	8.5	9.7	9.6	7.9	9.5	11.8	11.5	8.9
Limonene	1026	1024	0.3	0.2	0.3	0.3	0.2	0.2	0.2	0.2	0.2	0.3	0.2	0.2
1,8-Cineol	1032	1026	0.9	0.9	0.9	0.7	0.8	1.3	0.7	0.6	1.0	0.8	1.1	0.9
γ-Terpinene	1056	1054	1.3	2.0	1.6	1.7	1.7	0.2	1.7	1.5	0.1	1.9	1.1	0.8
Linalool	1098	1095	3.9	3.8	3.5	2.8	3.7	2.9	2.8	2.9	2.5	2.4	3.1	2.6
Ipsdienol	1144	1140	0.2	0.2	-	-	0.1	-	-	0.1	-	-	-	-
Umbellulone	1169	1167	0.1	0.3	0.2	0.3	0.3	0.3	0.4	0.5	0.1		0.3	0.3
Terpinen-4-ol	1176	1174	1.1	1.1	1.3	0.9	1.1	1	0.9	0.9	0.8	1.0	0.9	0.7
Thymol methyl ether	1234	1232	2.2	2	1.8	1.4	1.7	1.3	1.6	2.0	1.4	2.1	1.8	1.3
Thymol isomer (MW = 150)	1282	-	0.4	0.4	0.4	0.4	0.4	0.4	0.4	-	-	-	-	-
Thymol	1292	1289	9.0	10.3	12.5	11.7	11.7	12.8	11.8	11.5	9.8	9.3	8.2	9.2
Carvacrol	1299	1298	40.7	43.4	46.4	46.6	45.5	47.2	46.1	40.5	37.7	41.2	35.9	38.3
Thymol acetate	1351	1349	0.8	0.8	0.4	0.3	0.5	0.4	0.6	0.7	0.6	0.6	0.9	0.6
Carvacrol acetate	1372	1370	1.7	1.6	0.6	0.6	0.8	0.6	1.1	1.3	0.9	1.1	1.8	1.1
Geranyl acetate	1382	1379	0.4	0.4	0.3	0.3	0.4	0.6	0.4	0.6	0.6	0.5	0.7	0.8
(*E*)-Caryophyllene	1418	1416	3.4	3.5	2.5	2.6	3.4	2.3	2.1	2.8	2.7	2.9	4.8	4.6
*trans*-α-Bergamotene	1434	1432	0.2	0.2	0.2	0.2	0.2	0.2	0.1	0.2	0.2	-	0.2	0.2
α-Humulene	1455	1452	0.4	0.4	0.3	0.3	0.4	0.3	0.2	0.3	0.3	0.3	0.5	0.5
*p*-Methoxythymol	1487	1484	8.8	8.4	7.3	10.1	8.8	7.4	8.4	10.1	12.8	10.4	13.3	14.1
β-Bisabolene	1506	1505	0.3	0.4	0.3	0.3	0.4	0.3	0.2	0.4	0.4	0.3	0.4	0.5
(*Z*)-α-Bisabolene	1508	1506	-	0.2	0.2	0.2	0.2	0.2	-	0.3	0.4	0.6	0.5	0.3
δ-Cadinene	1524	1522	-	-	-	-	0.1	-	-	-	-	-	0.2	0.2
*trans*-γ-Bisabolene	1531	1529	0.4	0.5	0.4	0.5	0.6	-	0.5	-	-	-	-	0.7
*p*-Methoxycarvacrol (tent.)	1555	-	2.8	2.5	1.1	1.2	1.6	1.3	1.6	2.3	2.7	2	3.7	3.1
Caryophyllene oxide	1584	1582	3	1.8	1.3	1.6	1.7	1.6	2.2	2.8	2.8	3.6	2	1.9
2-phenylethyl tyglate	1587	1584	0.3	0.3	0.2	0.2	0.2	0.2	0.2	0.3	0.3	0.2	0.3	-
Humulene epoxide II	1611	1608	-	-	-	-	0.2	0.2	0.2	0.3	0.3	-	-	-
α-Cadinol	1655	1652	0.2	-	-	-	0.1	-	-	0.1	0.1	-	0.2	0.2
α-Eudesmol	1656	1653	-	-	-	-	0.1	0.1	-	0.1	0.2	-	0.2	0.2
α-Bisabolol	1686	1685	0.2	0.2	0.2	0.2	0.2	0.2	0.2	0.2	0.2	-	0.2	0.3
Unidentified sesquiterpenes	-	-	2.1	0.5	0.8	0.5	0.7	1.2	1.3	3.7	3.8	2.1	1.4	2.5
Total	-	-	97.8	99.0	98.8	99.0	98.7	96.1	97.7	93.1	92.0	96.1	98.2	95.9

RI_Calc_ = Retention Index on DB-5ms Column; RI_Lit_ = Retention index of literature (Adams, 2007) [23]. * Flowering period.

It is important to note that *Lippia origanoides* distributed by Central and South America also showed several chemotypes as mentioned previously. As in the present work, with oregano scent and the predominance of carvacrol in the oil, other specimens were identified in Piauí and Pará, Brazil, as well as in Santander, Colombia [2,3,7]. With also oregano smell, a thymol type was found in Merida, Venezuela, and regions of Cauca, Nariño and Boyacá, Colombia [7,8]. Another type with citric smell and significant content of α- and β-phellandrene, p-cymene, limonene and 1,8-cineole was also found in the Santander Region, Colombia [7]. *Lippia schomburgkiana*, a synonymous species of *L. origanoides*, with a fresh camphoraceous scent and existing in Maranhão, Brazil, showed another different type with the predominance of 1,8-cineole [9]. Recently, an oil of *L. origanoides* with occurrence in the Southern of Pará state, with fruity-woody odor and reminiscent of cinnamon, strawberry and wood, was reported [10]. The main constituents found in this oil were (*E*)-methyl cinnamate, (*E*)-nerolidol, *p*-cymene, 1,8-cineole, carvacrol, α-pinene, (*E*)-caryophyllene and γ-terpinene, showing a significant variation throughout the day and year. The authors have suggested that the environmental conditions of climate and the soil, which is covered by iron-manganese ore, could be contributing for this notable variation in oil composition. The temperature and relative humidity are lower in the Southern of Pará State. On the other hand, in the present specimen of *L. origanoides*, which was collected in Western Pará state, the oil composition did not vary significantly, which could be also attributed to the environmental conditions of climate and the soil, poor in mineral nutrients. The temperature and relative humidity are higher in Western Pará State.

### 2.3. Antimicrobial Activity of the Oil

The antimicrobial activity of *L. origanoides* oil on the bacteria *Staphylococcus aureus* and *Escherichia coli* was previously reported [2,3]. In the present study, with the same microorganisms, it was found that the antibacterial activity of *L. origanoides* was little influenced by the changed composition of the oil due to seasonal variation. For both bacteria, different MIC values were observed in the 12 months of data collection, as seen in Table 3. Against *S. aureus*, the MIC value was 1.25 μL/mL for the oils obtained within 10 months of the seasonal study, excepting March and May in which MIC value was 2.5 μL/mL. Against *E. coli*, the MIC value was 0.15 μL/mL in March, May, June, July, September and October, and 0.31 μL/mL in January, February, April, August, November and December. In all samples, the MBC values were 2.25 μL/mL and 1.25 μL/mL against *S. aureus* and *E. coli*, respectively.

**Table 3:** Antimicrobial potential of the essential oil of *L. origanoides* obtained in the seasonal study (January to December 2012).

Bacteria		Concentration (µL/mL)											
		Jan	Feb	Mar	Apr	May	Jun	Jul	Aug	Set	Oct	Nov	Dec
MIC	*Staphylococcus aureus*	1.25	1.25	2.25	1.25	2.25	1.25	1.25	1.25	1.25	1.25	1.25	1.25
	*Escherichia coli*	0.31	0.31	0.15	0.31	0.15	0.15	0.15	0.31	0.15	0.15	0.31	0.31
MBC	*Staphylococcus aureus*	2.25	2.25	2.25	2.25	2.25	2.25	2.25	2.25	2.25	2.25	2.25	2.25
	*Escherichia coli*	1.25	1.25	1.25	1.25	1.25	1.25	1.25	1.25	1.25	1.25	1.25	1.25

Studies have confirmed the influence of environmental factors as determinants of antimicrobial action of essential oils [35]. In the present work, no significant variation was observed in the antimicrobial activity when compared to seasonal collection period. Moreover, only low correlation was detected when analyzing the values obtained in the tests of antimicrobial activity in relation to the percentage contents of the major components present in the oils of seasonal study, as can be seen in Table 4.

**Table 4:** Simple correlation between antimicrobial activity and the main constituents (carvacrol and thymol) of the oil of *L. origanoides*.

Bacteria (MIC)	Correlation Coefficient (r^2^)	
	Carvacrol (%)	Thymol (%)
*Escherichia coli*	−0.411 *	−0.455 *
*Staphylococcus aureus*	0.413 *	0.442 *

* Significant at *p* ≤ 0.1.

The antimicrobial activity of the essential oil of *L. origanoides*, at least in part, must be associated with the presence of its major constituents, carvacrol and thymol [3,36]. These compounds exhibit high antimicrobial activity against different types of microorganisms and have been often studied in this context. Thus, Ultee and colleagues [37] have hypothesized that the hydroxyl group and the presence of a system of delocalized electrons are important for the antimicrobial activity of phenolic compounds, such as carvacrol and thymol. Furthermore, Ben Arja and colleagues [38] supported the hypothesis that the proton of the free hydroxyl is able to be distributed into the electronic system of the aromatic ring, enhancing the hydrophobic character of these compounds to allowing its accumulation in the cell membranes, causing its disintegration and death of the microorganisms. As for this biological action, it is clear that one cannot exclude the synergistic effect of the oil minor components.

Thus, based on the results of the seasonal study, the high yield of the oils analyzed, and the small variation in the content of carvacrol and thymol one could suggest that the antimicrobial activity identified in the aerial parts of *L. origanoides* will remain unchanged for the full year, allowing its medical use without any risk of loss or absence of any of the active principles of the plant.

## 3. Experimental Section

### 3.1. Plant Material

Leaves and thin stems (aerial parts) of *L. origanoides* were collected monthly from January to December 2012. Plant collection was performed in an experimental planting of the Federal University of Western Pará (UFOPA), identified as “Projeto Farmácia Viva” located on Highway Everaldo Martins, PA 457, Km 26, Municipality of Santarém, Pará State, Middle Amazon River, Brazil. The leaves and stems were sampled from clones of neighboring specimens, at the same growth stage, in order to minimize the phenological stage influence and the effects of light intensity, soil composition, and other environmental factors. The intermediate geographical position of the specimens was determined with GPS, resulting in the coordinates 02°30'870''S and 54°56'416''W, at an altitude of approximately 52 m above sea level. All collections were performed in the morning between 8 am and 10 am. Part of the collected material (180 g) was dried in an oven with air circulation (40 °C) until constant mass, whose data were used for calculation of the oil yield. Vouchers were deposited in the herbarium of Embrapa Amazônia Oriental (IAN-184688), Belém city, Pará state, and the herbarium of Federal University of Juiz de Fora (CESJ-64029), Juiz de Fora city, Minas Gerais state, Brazil.

### 3.2. Climatic Data

Climatic factors such as temperature, solar radiation, relative humidity and rain precipitation were measured monthly from January to December 2012. Data were obtained from a meteorological station installed near the experimental planting. The equipment used was: Datalogger Model CR1000 (Campbell, North Logan, UT, USA) Thermo-hygrometer model HMP45C (Vaisala, Ventura, CA, USA) Pyranometer model LI200 (LI-Cor, Lincoln, NE, USA) and a Rain Gauge Model TR-525 (Texas Electronic, Dallas, TX, USA).

### 3.3. Plant Processing and Extraction of the Essential Oils

The aerial parts of the plant (leaves and thin stems) samples were air-dried, ground and submitted to hydrodistillation using a Clevenger-type apparatus (180 g, 3 h). The oils were dried over anhydrous sodium sulfate, and their percentage contents were calculated on the basis of the plants dry weight. The moisture contents of the samples were calculated using a scale with moisture measurement by infra-red, after phase separation using a Dean-Stark glass trap (Sigma-Aldrich, St. Louis, MO, USA) (5 g, 60 min), and toluene as the solvent phase. The procedure was performed in triplicate.

### 3.4. Oil-Composition Analysis

The analysis of the oils was carried on Agilent Technology (Santa Clara, CA, USA) equipment consisting of a 6890 Plus Series GC coupled to a selective Mass Spectrometry Detector 5973 and an Auto Sampler 7863, under the following conditions: DB-5ms (60 m × 0.25 mm; 0.25 mm film thickness) fused-silica capillary column; programmed temperature, 50 °C (5 min in isothermal mode), 150 °C (4 °C/min plus 2 min in isothermal mode), 250 °C (5 °C/min plus 5 min in isothermal mode), 275 °C (10 °C/min plus 15 min in isothermal mode); injector temperature, 250 °C, injection type, split (1 μL) and split flow adjusted to yield 30:1 ratio; carrier gas, helium, with an inlet pressure of 16.5 psi; EIMS, electron energy, 70 eV; temperature of the ion source and transfer line, 230 and 285 °C, respectively. The quantitative data regarding the volatile constituents were obtained by peak-area normalization using a GC 6890 Plus Series coupled to FID Detector, operated under similar conditions of the GC-MS system. The retention index was calculated for all the volatiles constituents using a homologous series of C_8_–C_30_ *n*-alkanes (Sigma-Aldrich), according the linear equation of Van den Dool and Kratz [39].

### 3.5. Antimicrobial Bioassay

#### 3.5.1. Microorganisms and Inoculum Standardization

To monitor the variation of the antimicrobial potential of the *L. origanoides* oil, due to possible seasonal fluctuations, the tests were made by the broth micro dilution method, in triplicate [40]. Two strains of microorganisms were used: *Escherichia coli* ATCC 25922 (Gram-negative) and *Staphylococcus aureus* ATCC 25923 (Gram-positive). The microorganisms were commercially obtained in lyophilized form, rehydrated in nutrient broth (Becton/Difco, Franklin Lakes, NJ, USA) (24 h, 36 ± 1 °C) and then seeded in Petri dishes containing Mueller Hinton agar (Becton/Difco) (24 h, 36 ± 1 °C). The inocula (24 h) in sterile saline were prepared to achieve the turbidity standard of 0.5 in the McFarland scale, containing the suspensions approximately 1.5 × 10^8^ CFU/mL.

#### 3.5.2. Determination of Minimum Inhibitory Concentration (MIC) and Minimum Bactericidal Concentration (MBC)

In this method, the inoculum was standardized according to the scale of 0.5 MacFarland. It was subjected to serial dilution (saline solution) to obtain the final concentration of 1.5 × 10^4^ CFU/mL. The dilution of oil was done in solution of Tween 80 (1%) to obtain the test concentration (20 μL/mL) and then with serial dilution using Mueller-Hinton Broth (Becton/Difco), for the preparation of other concentrations. The tests were performed in 96 well plates, where each well received 90 μL of the specific concentration of the oil, 90 μL of Mueller Hinton broth (Himedia, Mumbai, India) and 20 μL of the inoculum. The control of microbial growth, control of sterility of the medium and the solvent control were done simultaneously. Each well had a final volume of 200 μL. The inhibition of growth of bacteria was revealed by addition of resazurin sterile solution (20 μL, 0.02%, w/v) (Vetec, São Paulo, Brazil), after incubation (24 h, 36 ± 1 °C). Then the plates were incubated for another 3 h. The MIC, which is defined as the lowest concentration of oil capable of inhibiting the growth of microorganisms, was determined by the permanence of blue coloration (resazurin) in the wells. The wells that showed no apparent growth were selected to evaluate the CBM, which was determined by the absence of microbial growth on plates containing agar MH [40].

### 3.6. Statistical Analysis

The climatic parameters, oil yield and antimicrobial activity were expressed as mean ± standard deviation. Statistical significance was evaluated using the Tukey’s test, considering as significant if *p* ≤ 0.05 (GraphPad Prism software, La Jolla, CA, USA, version 3.0, 2009). Pearson correlation coefficients were calculated using Minitab 14 (software, State College, PA, USA, version 14, 2011) to determine the relationship between the analyzed parameters.

## 4. Conclusions

The results showed that the seasonal variation in 12 months did not affect the average yield of the essential oil of *L. origanoides*, and also has little influence on its composition and antimicrobial potential. Thus, the information gathered during the course of this study extends the knowledge of this species with significant medicinal potential, furnishing scientific data that can be of great interest to the phyto-pharmaceutical industry in the future.
